# Simple, streamlined, cost-effective cDNA synthesis method from cell cultures

**DOI:** 10.1098/rsob.240226

**Published:** 2025-03-12

**Authors:** Daniel Stránský, Monika Šteigerová, Markéta Kuklová, Veronika Hanzíková, Nikolina Canová, Jiří Novotný, Ladislav Šenolt, Ondřej Slanař

**Affiliations:** ^1^ Department of Pharmacology, First Faculty of Medicine, Charles University, Praha, Czech Republic; ^2^ Department of Pharmacology, General University Hospital, Praha, Czech Republic; ^3^ Department of Rheumatology, Institute of Rheumatology, Praha, Czech Republic; ^4^ Faculty Transfusion Center, General University Hospital, Praha, Czech Republic; ^5^ Department of Physiology, Faculty of Science, Charles University, Praha, Czech Republic

**Keywords:** peripheral blood mononuclear cells, RNA isolation, mRNA, qPCR, *in vitro*, cell lysis, proteinase k

## Abstract

Applications like drug development need simple and streamlined methods to process samples from 96-well cell culture plates for gene expression measurements. Unfortunately, current options are expensive for such processing. Therefore, our aim was to develop a method that would allow streamlined analysis of mRNA from 96-well cell culture plates while being relatively cheap and simple. We developed a method based on the qPCR ‘Cells-to-cDNA’ approach and validated it against commercially available kits using the same approach or spin columns-based RNA purification. For this purpose, we conducted a series of comparisons of gene expression from peripheral blood mononuclear cells, SK-HEP-1 and U-87 cell cultures in 96-well plates. Our final method involved lysing cells with 25–100 µl solution of 0.5% SDS, 10 mM DTT, 1 mg ml^−1^ proteinase K dissolved in water, 1 h incubation at 50°C, followed by proteinase K inactivation at 90°C for 5 min and lysate neutralization with 1 : 1 dilution by 20% Tween 20 solution. Reverse transcription and qPCR were carried out using standard methods. This method showed a mean reduction of Ct ± s.d. value by 2.4 ± 1.3 compared with the ‘Cells-to-cDNA’ kit and by 1.4 ± 0.5 compared with the RNA purification kit with lower variability.

**Figure:**
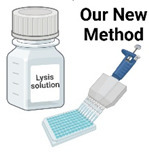

## 1. Introduction

Understanding gene expression is essential for a better understanding of physiological and pathophysiological processes, which is why measurement of mRNA expression has become widely used. The mRNA expression analyses are based on methods such as northern blotting, qPCR, microarrays and RNA sequencing [1]. Northern blot is the oldest, cheapest and most simple method, but its sensitivity is low compared with other methods [1,2]. Additionally, only a few samples can be processed at any given time, and it is labour-intensive. Microarray and RNA-seq methods enable streamlined gene analysis but are relatively expensive (especially RNA-seq), require specialized equipment and software and are relatively time-consuming [1,3,4]. Furthermore, microarray assays have low accuracy and specificity compared with other methods. Therefore, qPCR-based assays are usually considered the gold standard method due to their relative affordability and simplicity, while remaining accurate and sensitive. There are two main approaches to qPCR-based methods. The first involves isolating and purifying RNA, followed by conversion to cDNA by reverse transcription. The second approach, sometimes called the ‘Cells-to-cDNA’ method, skips the isolation and purification step by directly reverse transcribing RNA from cell/tissue lysates. This method can be either two-step, where reverse transcription and cDNA amplification are separate steps, or one-step with both processes occurring simultaneously in a single reaction. For the analysis of mRNA from whole 96-well plates, RNA can be purified using filter-based spin columns or magnetic beads or the aforementioned ‘Cells-to-cDNA’ method may be suitable. These methods are available as commercially available kits, however, their advantage of processing an entire 96-well plate at once is counterbalanced by the high cost, typically no less than €1000 per 96-well plate. Therefore, our aim was to develop a simple, streamlined and cost-effective method for analysing mRNA from 96-well cell culture plates that is sufficiently accurate and sensitive. Considering these factors, we decided to base our method on the ‘Cells-to-cDNA’ approach.

## 2. Material and methods

### 2.1. Donors

Anonymized blood samples from healthy donors were obtained from the Blood Transfusion Department of the General University Hospital in Prague, Czech Republic. The blood donation process for this research was reviewed and approved by the Ethics Committee of the General University Hospital, Prague, Czech Republic under the no. 169/23 S-IV. All participants provided written informed consent.

### 2.2. Cell cultures

Peripheral blood mononuclear cells (PBMC) were isolated by standard density gradient method using Ficoll-Paque® Plus (Sigma-Aldrich; GE17-1440-03). The U-87 MG (human glioblastoma astrocytoma) cell line (cat. no. 89081402) and the SK-HEP-1 (human hepatic adenocarcinoma) cell line (cat. no. 91091816) were purchased from the European Collection of Authenticated Cell Cultures (ECACC; Culture Collections UK Health Security Agency, Porton Down, Salisbury, UK). U-87 and SK-HEP-1 cells were cultured in cell culture flasks to 80% confluence in a CO_2_ incubator under standard conditions (37°C, 5% CO_2_ and a humidified atmosphere) prior to counting and seeding. Cells were counted in LUNA-FX7^TM^ automated cell counter (Logos Biosystems, Canada) using the fluorescent dye and seeded into the 96-well culture plate to achieve a final volume of 100 µl per well and a density of 10^6^ cells ml^−1^, 2 × 10^5^ cells ml^−1^ and 10^5^ cells ml^−1^ for PBMC, U-87, and SK-HEP-1, respectively. The composition of culture medium for PBMC was as follows: RPMI-1640 (Sigma-Aldrich; R8758-6X500 ML) with 2 mM L-glutamine, 100 μg ml^−1^ streptomycin, 100 IU ml^−1^ penicillin (Sigma-Aldrich; P4333), 10% FBS (Sigma-Aldrich; F9665-500ML) and 25 mM HEPES (Sigma-Aldrich; H3537-100ML). For U-87 and SK-HEP-1 cells, the cell culture medium (Dulbecco’s modified Eagle's medium: high glucose; Sigma-Aldrich, D6429-500ML) was supplemented with 10% FBS (Sigma-Aldrich; F9665-500ML), 100 μg ml^−1^ streptomycin and 100 IU ml^−1^ penicillin (Sigma-Aldrich; P4333). Cells were incubated in the atmosphere containing 5% of CO_2_. Cells were pre-incubated for 1 h with either control solution (0.5% DMSO solution in culture medium) or with 100 µM capsaicin (MedChemExpress; HY-10448) as a pro-inflammatory stimulant or with 100 µM hydroxychloroquine (MedChemExpress; HY-B1370) as an anti-inflammatory agent. Each group consisted of six wells. After the pre-incubation, all wells except the negative controls were stimulated with LPS from *Escherichia coli* O55:B5 (Sigma-Aldrich; L6529) in the final concentration of 100 ng ml^−1^ or TNFα (Thermo Fisher Scientific; PHC3011) in final concentrations of 1, 10 or 100 ng ml^−1^. After 6 h of incubation, cultivation was terminated, and cells were further processed for cDNA synthesis either by our newly developed method or by using the commercially available kit.

#### 2.2.1. Fibroblast whole 96-well plate culture

Fibroblasts (Lonza; CC-2511) were cultured in cell culture flasks to 80% confluence in a CO_2_ incubator under standard conditions (37°C, 5% CO_2_ and a humidified atmosphere) prior to counting and seeding. Cells were counted in LUNA-FX7^TM^ automated cell counter (Logos Biosystems, Canada) using the fluorescent dye and seeded into all 96 wells of the 96-well culture plate to achieve a final volume of 100 µl per well and density of 4 × 10^4^ cells ml^−1^. The composition of the culture medium was the same as for PBMC. For 24 h cells were incubated in the atmosphere containing 5% of CO_2_ and after this period cells were further processed for cDNA synthesis by our newly developed method.

### 2.3. Newly developed method for cDNA preparation

**Figure 1.**
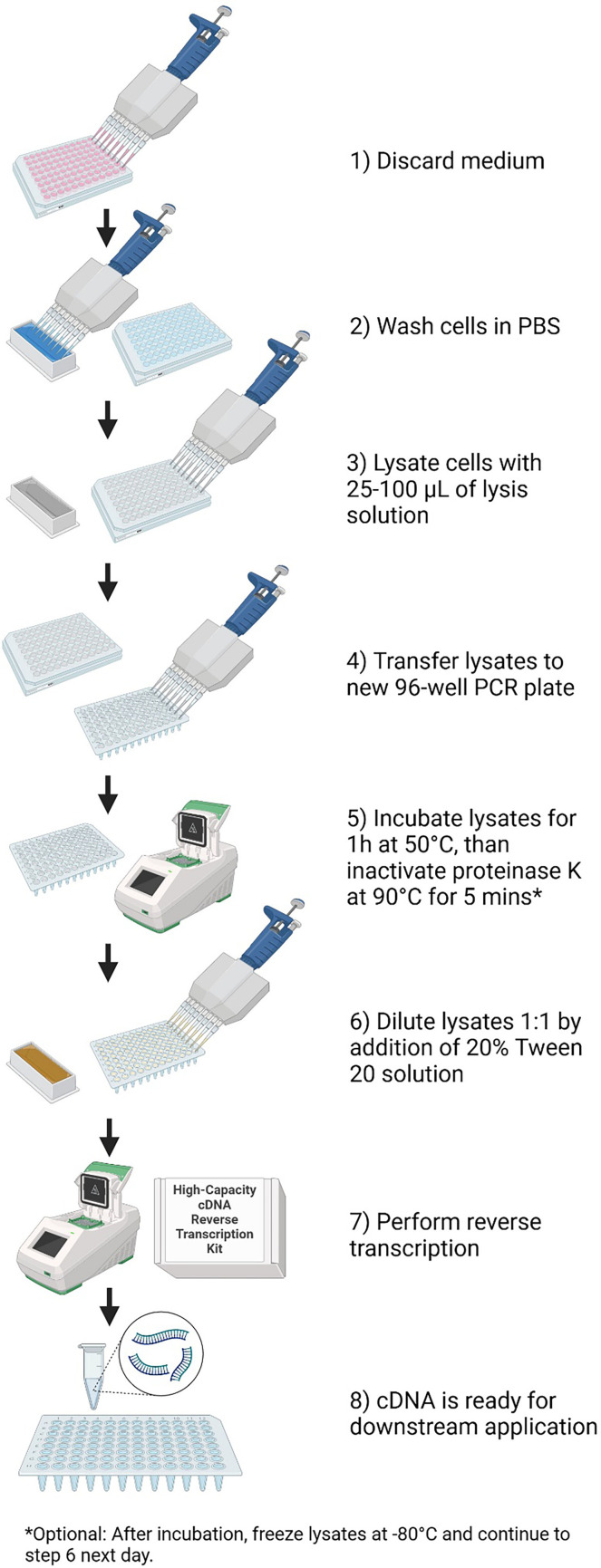
Simplified workflow of our new method. Created with BioRender.com.

The following final protocol was developed after a series of optimization experiments: culture plates were centrifuged at 100–1000*g* for 1–5 min (number of plates and time depending on the cell type) and placed on ice. After centrifugation, the medium was discarded, and cells were washed by icecold (4°C) PBS. Cells were then re-pelleted by another centrifugation at 100–1000*g* for 1–5 min and after centrifugation, PBS was discarded. Cells were then lysed while keeping them on ice in 25–100 µl (volume of the lysis solution depends on the number of cells per well and cell type) of our lysis solution with final composition of 0.5% SDS (Sigma-Aldrich; 71736-100 ML), 10 mM OmniPur® DTT (Sigma-Aldrich; 3860-OP), 1 mg ml^−1^ proteinase K (Thermo Fisher Scientific; AM2546) dissolved in PCR grade water. Lysates were transferred to a 96-well PCR plate and incubated in MyCycler™ Thermal Cycler (Bio Rad Laboratories, USA) for 1 h at 50°C followed by proteinase K inactivation at 90°C for 5 min. Form lysates of 20 µl were transferred to a new 96-well PCR plate and were diluted 1 : 1 by the addition of 20 µl of 20% Tween 20 solution. This mix was used for cDNA synthesis by the High-Capacity cDNA Reverse Transcription Kit (Thermo Fisher Scientific; 4368813) according to the manufacturer’s instructions (10 µl sample + 10 µl reverse transcription master mix). The simplified workflow is shown in figure 1.

### 2.4. Comparison with reference methods

**Figure 2.**
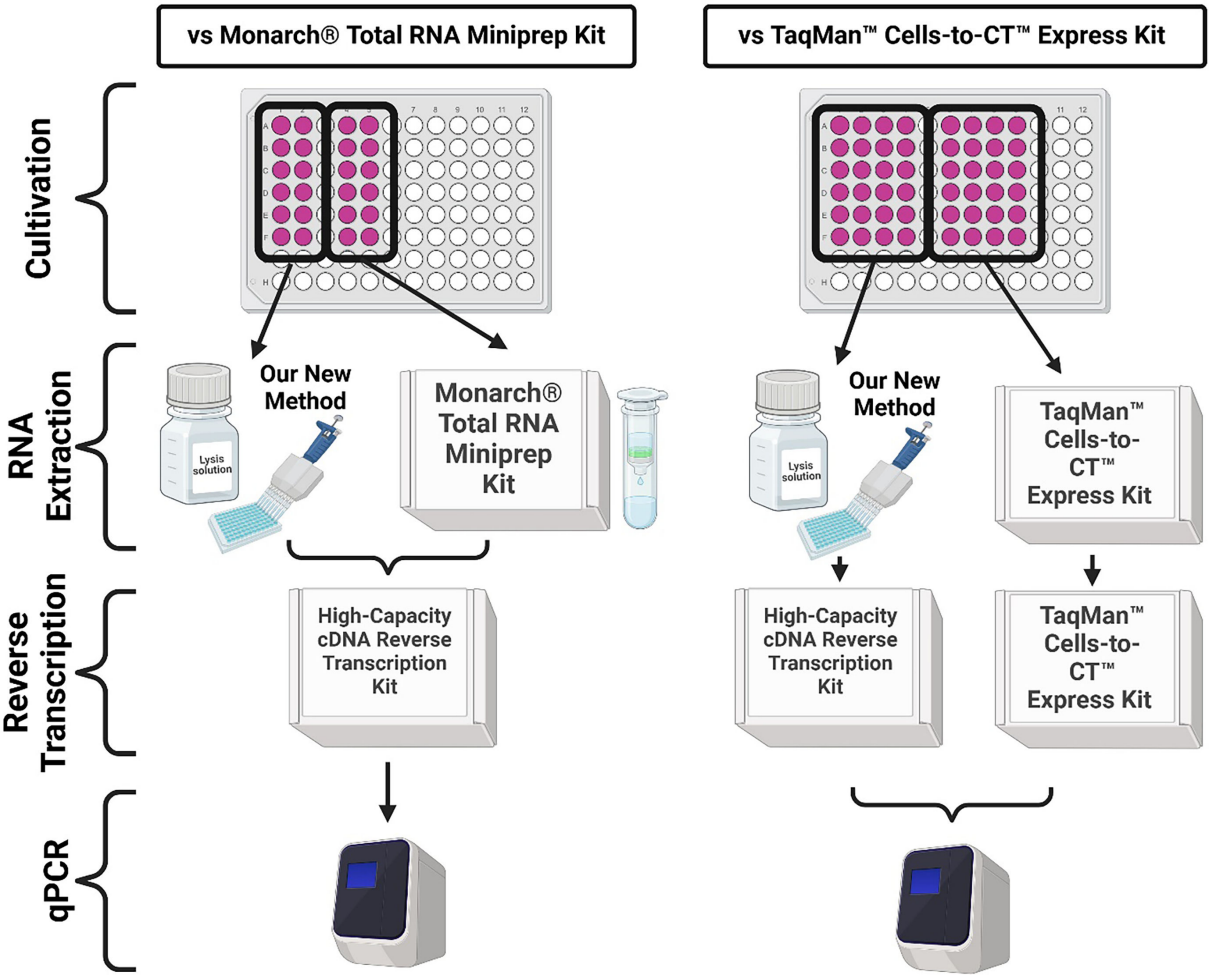
Method comparison workflow. Created with BioRender.com*.*

The TaqMan™ Cells-to-CT™ Express Kit (Thermo Fisher Scientific; A57985) and Monarch® Total RNA Miniprep Kit (New England Biolabs, USA) were used as reference methods for mRNA extraction and cDNA synthesis to compare the performance of our new method. The kits were used according to the manufacturers’ instructions after the PBS wash step simultaneously with our new method by another researcher. As we were not using DNase treatment in our method, we decided not to use DNase treatment with reference methods either. On the recommendation of the manufacturer for the Monarch® Total RNA Miniprep Kit, 100 µl of elution solution was used to provide the highest RNA yield. On the recommendation of the manufacturer for the TaqMan™ Cells-to-CT™ Express Kit, 50 µl of lysis solution was used and 10 µl of this lysate was further used in the reverse transcription mix with 40 µl of the reverse transcription master mix to provide the best results. The identical High-Capacity cDNA Reverse Transcription Kit (Thermo Fisher Scientific; 4368813) used in our new method was also used for cDNA synthesis when using the Monarch® Total RNA Miniprep Kit. The diagram describing how our methods compare is shown in figure 2.

### 2.5. RNA integrity and cDNA product stability

**Figure 3.**
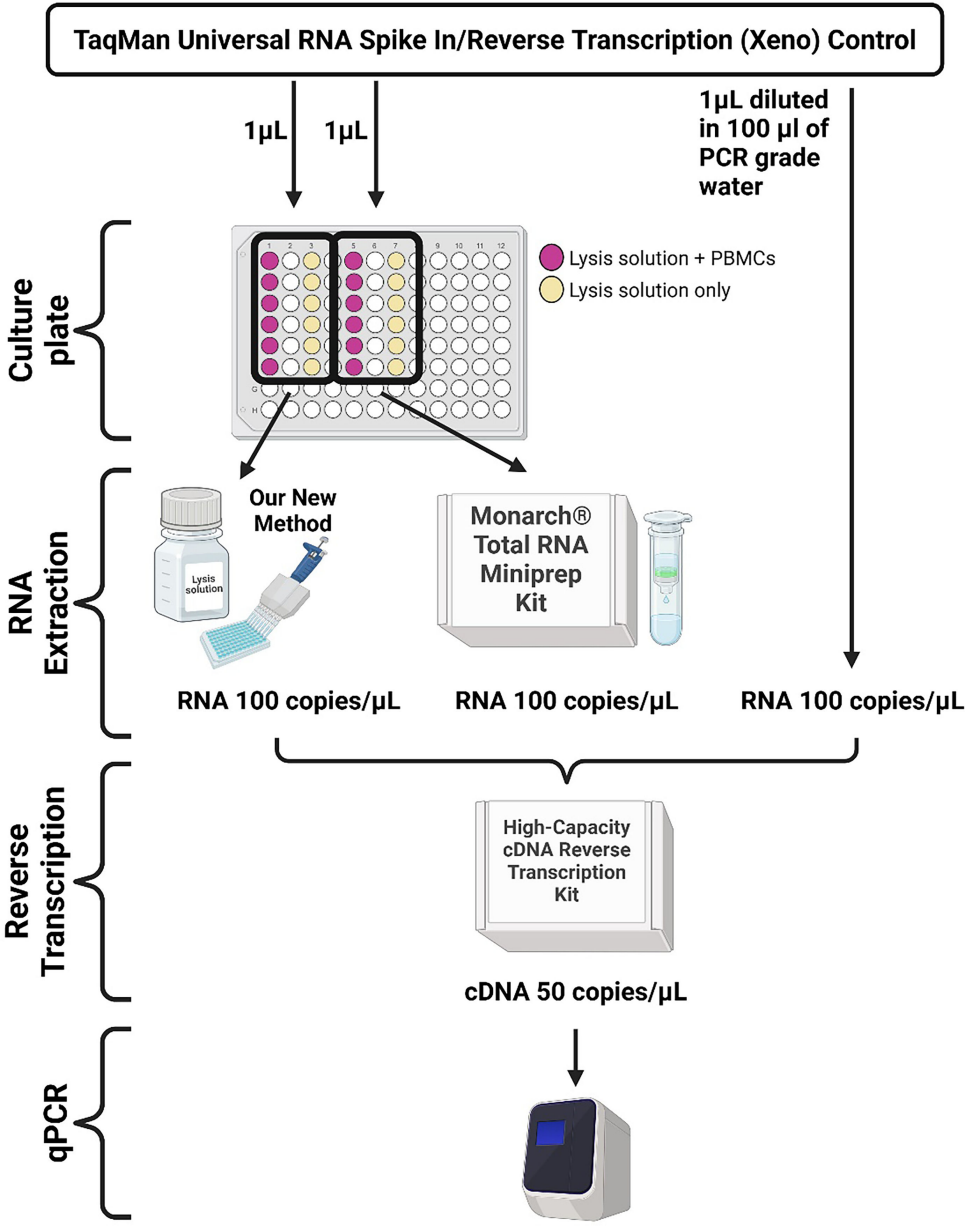
RNA integrity workflow. Created with BioRender.com.

The integrity of RNA was tested using commercially available TaqMan Universal RNA Spike In/Reverse Transcription (Xeno) Control (Thermo Fisher Scientific; A39179). This RNA is supplied in a known amount of 10 000 copies μl^−1^. To our lysis solution of 50 µl, 1 µl was added either alone or with PBMC in the amount of 10^5^ cells per well. As a reference, 1 µl was also added to the lysis solution of the Monarch® Total RNA Miniprep Kit either alone or with PBMC in the amount of 10^5^ cells per well with a final elution volume of 100 µl. After dilution of 1 : 1 with Tween 20 in our new method, both our new method and the reference method had the same concentration of 100 copies µl^−1^ of RNA spike before the cDNA synthesis step. With the addition of 10 µl of sample amount to 20 µl reaction volume in reverse transcription using the High-Capacity cDNA Reverse Transcription Kit (Thermo Fisher Scientific; 4368813) the final concentration of RNA spike and thus cDNA should be 50 copies µl^−1^ (1000 copies 20 µl^−1^). Additionally, the RNA spike was added directly to reverse the transcription reaction in a concentration matching our new method and reference kit. For the resulting cDNA qPCR amplification, predesigned matching TaqMan® Assay for Xeno™ sequences (Thermo Fisher; Assay ID Ac00010014_a1) were used together with complementary TaqMan™ Fast Advanced Master Mix (Thermo Fisher Scientific; 4444963). The diagram describing RNA integrity access is shown in figure 3.

Storage stability of the cDNA was verified after one month of storage at 20°C by repeating qPCR of RNA spike cDNA.

### 2.6. DNase treatment

To test the compatibility of DNase treatment with our new method, an additional DNase treatment step was added before cDNA synthesis for PBMC cells in the unstimulated group using DNase I (Thermo Fisher Scientific; EN0521). The reaction was carried out as follows: 1 µl of DNase I together with 1 µl of supplied reaction buffer was added to 8 µl of cell lysate after dilution with Tween 20. This mixture was incubated for 30 min at 37°C with subsequent DNase inactivation by the addition of 1 µl of supplied EDTA solution and incubation at 65°C for 10 min. The functionality of this DNase treatment was tested by measuring the expression of the PPIA (see table 1) gene with probe assay amplifying gDNA. The effect of DNase treatment on RNA stability was tested by standard gene expression measurement with cDNA-specific assay in the DNase-treated group and no DNase treatment group.

**Table 1.** List of used IDT assay with assay numbers and their target genes.

official gene symbol	official full gene name	IDT assay no.
IL6	interleukin 6	Hs.PT.58.40226675
NFKB1	nuclear factor kappa B subunit 1	Hs.PT.58.1434854
STAT3	signal transducer and activator of transcription 3	Hs.PT.58.20367494
TBP	TATA-box binding protein	Hs.PT.58.20792004
HPRT1	hypoxanthine phosphoribosyltransferase 1	Hs.PT.39a.22214821
B2M	beta-2-microglobulin	Hs.PT.58v.18759587
YWHAZ	tyrosine 3-monooxygenase/tryptophan 5-monooxygenase activation protein zeta	Hs.PT.39a.22214858
PPIA	peptidylprolyl isomerase A	Hs.PT.58v.38887593.g

### 2.7. Quantitative PCR

For cDNA qPCR amplification, predesigned probe-based assays (Integrated DNA Technologies, Coralville, USA) (table 1) were used together with complementary TaqMan™ Fast Advanced Master Mix (Thermo Fisher Scientific; 4444963). The reaction was carried out in QuantStudio™ 3 Real-Time PCR System (Thermo Fisher Scientific, USA) with reaction parameters set up according to the reagent manufacturer’s recommendation. All qPCRs were run with no-reverse transcriptase control and negative control. Predesigned probe-based assays were tested beforehand for their cDNA specificity. These formulas were used to calculate ∆∆Ct and RQ:

∆∆Ct = ∆Ct (non-stimulated group) – ∆Ct (stimulated group); when ∆Ct = Ct of gene of interest (IL6, NFKB1, STAT3) – mean Ct of two housekeeping genes (TBP, HPRT1)


$$RQ=2^{-(∆∆Ct)}$$

### 2.8. One day delayed reverse transcription

Samples from non-stimulated PBMC cells were frozen after step 5 and step 6 in our simplified protocol (figure 1) to test if reverse transcription could be postponed until the next day.

### 2.9. Data analysis

Descriptive statistics and Student’s *t*‐test have been processed in GraphPad Prism 8.0.1 (GraphPad Software, Inc., La Jolla, USA).

## 3. Results

### 3.1. RNA integrity and cDNA product stability

**Figure 4.**
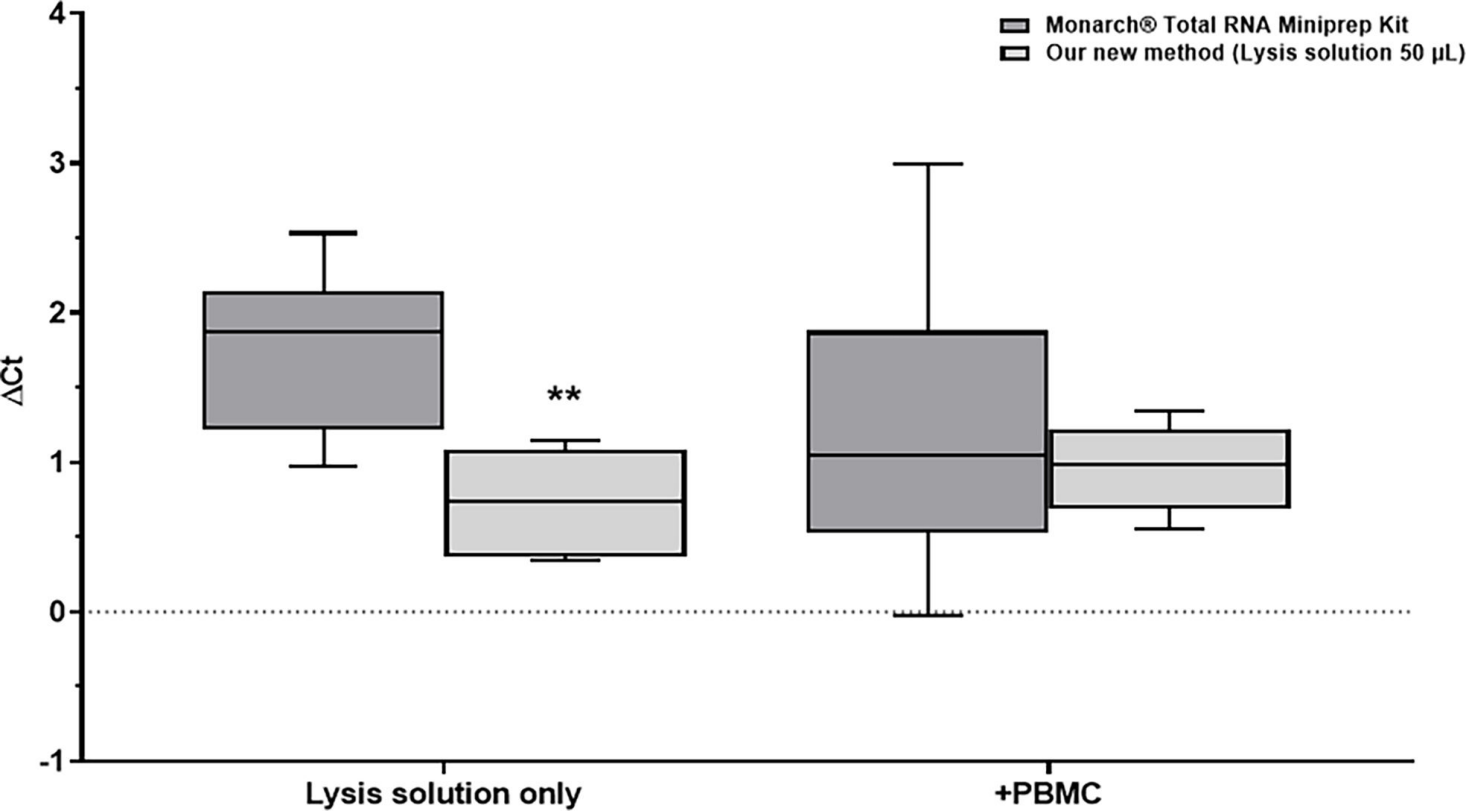
RNA integrity comparison. Data are expressed as mean ± s.d. (*n* = 6). ΔCt = Ct value of RNA spike isolated using our new method or reference method (Monarch® Total RNA Miniprep Kit) – Ct value of RNA spike directly added to reverse transcription. Lysis solution only: ΔCt value of RNA spike isolated from lysis solution only (no cells included). +PBMC – ΔCt value of RNA spike isolated from cell lysates of PBMC 10^5^ cells per well. Light grey columns: our new method using 50 µl of lysis solution; dark grey columns: Monarch® Total RNA Miniprep Kit. **p ≤ 0.01.

The expression of RNA spike resulted in the mean ± s.d. Ct values of 30.8 ± 0.3, 30.6 ± 0.3, 31.1 ± 0.9 and 31.6 ± 0.5 were obtained for our new method in cell lysates, lysis solution only, the reference method in cell lysates and lysis solution only, respectively. In comparison, the Ct value of the RNA spike directly added to reverse transcription (RNA control) was 29.9 ± 0.3. Subtracting this value from the Ct values obtained with both methods resulted in mean ± s.d. ΔCt value of 1 ± 0.3, 0.7 ± 0.3, 1.2 ± 0.9 and 1.8 ± 0.5 for our new method in cell lysates, lysis solution only, reference method in cell lysates and lysis solution only, respectively (figure 4). Our new method showed significantly lower mean ± s.d. ΔCt value compared with the reference method in lysis solution only (0.7 ± 0.3 versus 1.8 ± 0.5, respectively; *p* ≤ 0.01) but no significant difference in mean ± s.d. ΔCt value in cell lysates 1 ± 0.3 versus 1.2 ± 0.9, respectively; *p* = 0.6).

**Figure 5.**
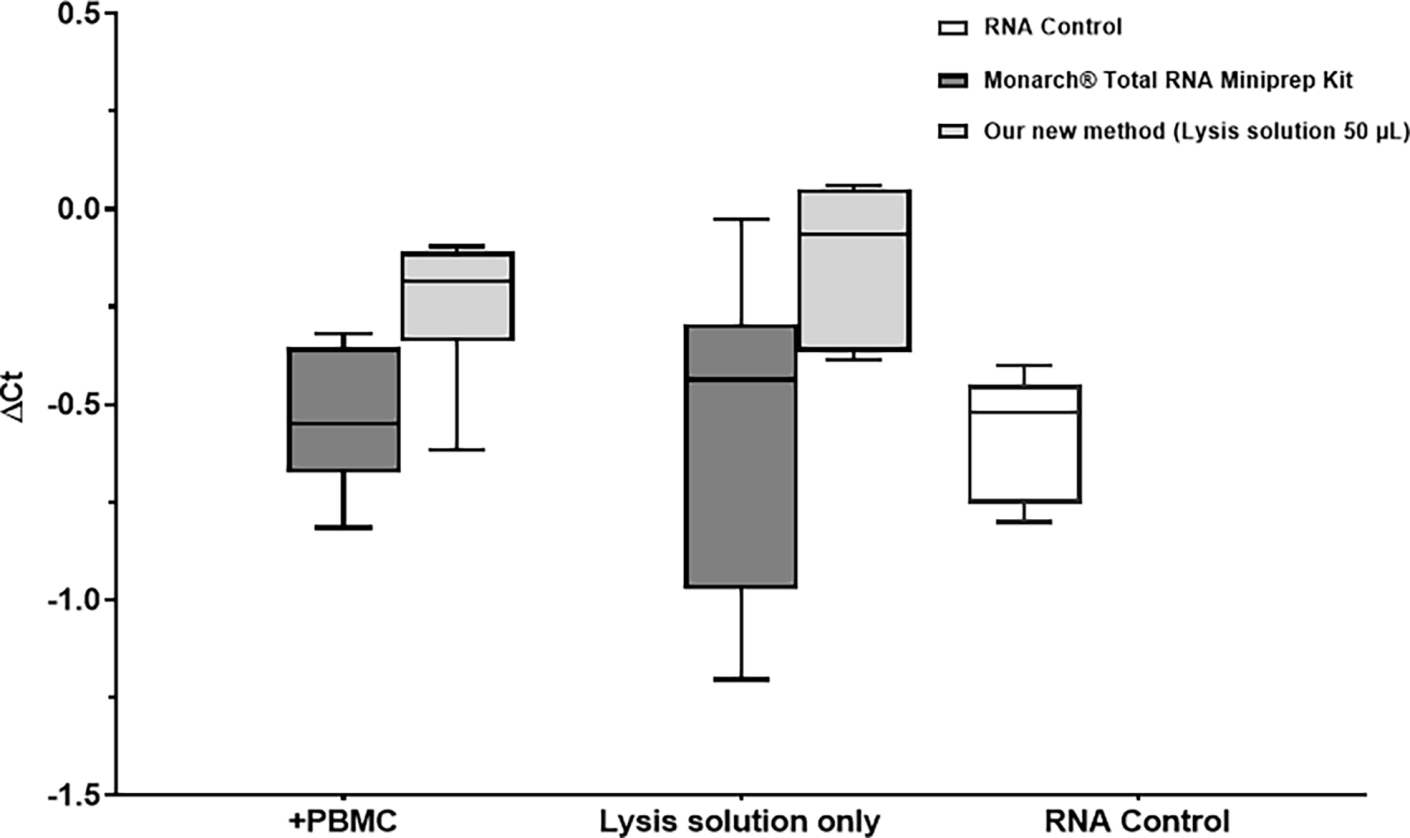
cDNA stability comparison. Data are expressed as mean ± s.d. (*n* = 6). ΔCt = Ct value measured immediately after cDNA synthesis − Ct value measured after one month of storage at −20°C. Lysis solution only: ΔCt value of RNA spike isolated from lysis solution only (no cells included). +PBMC − ΔCt value of RNA spike isolated from cell lysates of PBMC 10^5^ cells per well. RNA control − ΔCt of RNA spike directly added to reverse transcription. Light grey columns: our new method using 50 µl of lysis solution; dark grey columns: Monarch® Total RNA Miniprep Kit.

The stability of cDNA synthesized from the RNA spike was tested by repeating qPCR after one month of storage at −20°C. Subtracting each Ct value before and after one month resulted in mean ± s.d. ΔCt of −0.2 ± 0.2, −0.1 ± 0.2, −0.5 ± 0.2, −0.6 ± 0.4 and −0.6 ± 0.1 for our new method in cell lysates, lysis solution only, reference method in cell lysates, lysis solution only and RNA spike directly added to reverse transcription (RNA control), respectively (figure 5).

### 3.2. Comparison with RNA purification based method

When comparing gene expression data obtained with the Monarch® Total RNA Miniprep Kit, our new method showed lower mean ±s.d. Ct values when measuring NFKB1, STAT3, IL6, TBP and HPRT1 from non-stimulated and stimulated PBMC, U-87 and SK-HEP-1 cells (table 2). In total, in 29 out of 30 comparisons conducted our new method showed significantly lower Ct value than the reference method. The mean ± s.d. difference between the Ct value of the reference method and our new method across all genes and cell types was 1.4 ± 0.5 (1.2 ± 0.5, 1.2 ± 0.4 and 1.9 ± 0.3 for PBMC, U-87 and SK-HEP-1 cells, respectively). There was visibly reduced variability with the mean ± s.d. CV% of 0.99 ± 0.35% versus 1.26 ± 0.42% (*p* ≤ 0.01) in our new method versus the reference method, respectively.

**Table 2.** Comparison of Ct values of NFKB1, STAT3, IL6, TBP and HPRT1 genes obtained by our new method (PBMC, 50 µl of lysis solution; U-87, 25 µl of lysis solution; SK-HEP-1, 25 µl of lysis solution) and the Monarch® Total RNA Miniprep Kit from PBMC, U-87 and SK-HEP-1 cells. Data are expressed as means ± s.d. (*n* =6). NEG, negative (non-stimulated).

group	gene	cell type	ONM Ct mean ± s.d.	RM Ct mean ± s.d.	*p-*value versus ONM	cell type	ONM Ct mean ± s.d.	RM Ct mean ± s.d.	*p*-value versus ONM	cell type	ONM Ct mean ± s.d.	RM Ct mean ± s.d.	*p*- value versus ONM
NEG	IL6	PBMC	34.3 ± 0.3	36.6 ± 0.8	*p* < 0.001	U-87	25.9 ± 0.3	26.8 ± 0.3	*p* < 0.001	SK-HEP-1	25.8 ± 0.4	27.2 ± 0.4	*p* < 0.001
	NFKB1		28.4 ± 0.3	29.9 ± 0.3	*p* < 0.001		28.9 ± 0.3	30.2 ± 0.3	*p* < 0.001		26.8 ± 0.5	28.8 ± 0.3	*p* < 0.001
	STAT3		26 ± 0.3	27.1 ± 0.3	*p* < 0.001		25.1 ± 0.3	27 ± 0.3	*p* < 0.001		23.2 ± 0.3	25.5 ± 0.4	*p* < 0.001
	TBP		30.5 ± 0.3	31.6 ± 0.4	*p* < 0.001		27 ± 0.2	28.2 ± 0.3	*p* < 0.001		26.5 ± 0.4	28.5 ± 0.5	*p* < 0.001
	HPRT1		31 ± 0.3	32.2 ± 0.6	*p* < 0.001		25 ± 0.2	27 ± 0.3	*p* < 0.001		23.9 ± 0.1	26 ± 0.4	*p* < 0.001
STIM	IL6		24.2 ± 0.1	24.4 ± 0.2	*p* = 0.1		23.3 ± 0.3	24 ± 0.2	*p* < 0.001		24.4 ± 0.4	25.6 ± 0.3	*p* < 0.001
	NFKB1		26.4 ± 0.2	27.6 ± 0.7	*p* < 0.001		28.1 ± 0.2	28.9 ± 0.3	*p* < 0.001		25.7 ± 0.2	27.6 ± 0.4	*p* < 0.001
	STAT3		24.8 ± 0.2	25.7 ± 0.2	*p* < 0.001		25.4 ± 0.3	26.5 ± 0.2	*p* < 0.001		23.2 ± 0.2	25.1 ± 0.4	*p* < 0.001
	TBP		30.3 ± 0.1	31.4 ± 0.1	*p* < 0.001		26.7 ± 0.1	27.8 ± 0.2	*p* < 0.001		26.2 ± 0.3	28.3 ± 0.4	*p* < 0.001
	HPRT1		30.4 ± 0.2	31.8 ± 0.3	*p* < 0.001		25.4 ± 0.3	26.6 ± 0.2	*p* < 0.001		24.2 ± 0.2	26 ± 0.4	*p* < 0.001

Regarding ΔΔCt and RQ values both methods showed comparable results (table 3). There were only two comparisons, where both methods differed (STAT3 gene in U-87 cells and SK-HEP-1 cells) with the difference in RQ of ≤0.2, therefore we do not consider this to have any practical significance.

**Table 3.** Comparison of ΔΔCt values and RQ values of NFKB1, STAT3 and IL6 genes obtained by our new method (PBMC, 50 µl of lysis solution; U-87, 25 µl of lysis solution; SK-HEP-1, 25 µl of lysis solution) and Monarch® Total RNA Miniprep Kit from PBMC, U-87 and SK-HEP-1 cells. Data are expressed as means ± s.d. (*n* =6). NEG negative (non-stimulated) control; CTR100, control stimulated with LPS 100 ng ml^−1^; TNF10 control stimulated with TNFα 10 ng ml^−1^. ONM, our new method, RM, reference method (Monarch® Total RNA Miniprep Kit).

cell type	group	gene	ONM ΔΔCT mean ± s.d.	*p*-value versus ONM NEG	ONM RQ	RM ΔΔCT mean ± s.d.	*p*-value versus RM NEG	RM RQ
PBMC	CTR100	IL6	−9.7 ± 0.3	*p* < 0.001	856.4	−12 ± 1.1	*p* < 0.001	6026.4
		NFKB1	−1.6 ± 0.2	*p* < 0.001	3.1	−2 ± 0.8	*p* < 0.001	4.7
		STAT3	−0.8 ± 0.2	*p* < 0.001	1.8	−1 ± 0.2	*p* < 0.001	2.1
U-87	TNF10	IL6	−2.6 ± 0.3	*p* < 0.001	6	−2.4 ± 0.1	*p* < 0.001	5.3
		NFKB1	−0.9 ± 0.2	*p* < 0.001	1.9	−0.8 ± 0.1	*p* < 0.001	1.8
		STAT3	0.3 ± 0.1	*p* < 0.001	0.8	−0.1 ± 0.1	*p* = 0.09	1.1
SK-HEP-1	TNF10	IL6	−1.4 ± 0.3	*p* < 0.001	2.6	−1.4 ± 0.1	*p* < 0.001	2.7
		NFKB1	−1.1 ± 0.3	*p* < 0.001	2.2	−1.1 ± 0.1	*p* < 0.001	2.1
		STAT3	0 ± 0.2	*p* = 0.9	1	−0.2 ± 0.1	*p* < 0.001	1.2

### 3.3. Comparison with cells-to-cDNA method

When comparing gene expression data obtained with the TaqMan™ Cells-to-CT™ Express Kit, our new method showed lower mean ± s.d. Ct values when measuring NFKB1, STAT3, IL6, TBP and HPRT1 from PBMC (table 4), U-87 (table 5) and SK-HEP-1 (table 6) cells. In total, in 53 out of 60 comparisons our new method showed significantly lower Ct value than the reference method. The mean ± s.d. difference between the Ct value of the reference method and our new method across all genes and cell types was 2.4 ± 1.3 (2.9 ± 1.1, 1.5 ± 1.1 and 2.6 ± 1.2 for PBMC, U-87 and SK-HEP-1 cells. respectively). There was visibly reduced variability with the mean ± s.d. CV% of 1.05±0.55% versus 1.59±0.68% (*p* ≤ 0.001) in our new method compared with the reference method, respectively.

**Table 4.** Ct values of NFKB1, STAT3, IL6, TBP and HPRT1 genes obtained by our new method (100 µl of lysis solution) compared to TaqMan™ Cells-to-CT™ Express Kit from PBMC cells. Data are expressed as means ± s.d. (*n* =6). NEG: negative (non-stimulated); CTR100, control stimulated with LPS 100 ng ml^−1^; CAP100, cells preincubated with 100 µM of capsaicin; HCQ 100, cells pre-incubated with 100 µM of hydroxychloroquine. ONM, our new method, RM, reference method (TaqMan™ Cells-to-CT™ Express Kit).

PBMC		ONM Ct mean ± s.d.	RM Ct mean ± s.d.	*p-* value versus ONM	ONM Ct mean ± s.d.	RM Ct mean ± s.d.	*p*- value versus ONM	ONM Ct mean ± s.d.	RM Ct mean ± s.d	*p*- value versus ONM	ONM Ct mean ± s.d.	RM Ct mean ± s.d.	*p*- value versus ONM	
group:		NEG			CTR100			CAP100			HCQ			
gene	IL6	34 ± 1.2	35.2 ± 0.5	*p* = 0.075	26.3 ± 0.3	28.4 ± 0.5	*p* < 0.001	25 ± 0.3	28.1 ± 0.9	*p* < 0.001	29.8 ± 0.5	30.8 ± 0.4	*p* < 0.01	
	NFKB1	28.5 ± 0.6	33.4 ± 0.7	*p* < 0.001	26.7 ± 0.3	31.3 ± 0.4	*p* < 0.001	26.7 ± 0.2	31.8 ± 0.7	*p* < 0.001	27.9 ± 0.5	31.1 ± 0.4	*p* < 0.001	
	STAT3	26.8 ± 0.5	29.8 ± 0.4	*p* < 0.001	25.6 ± 0.3	28.7 ± 0.1	*p* < 0.001	26 ± 0.2	29.1 ± 0.4	*p* < 0.001	26.5 ± 0.5	28.4 ± 0.2	*p* < 0.001	
	TBP	29 ± 0.5	32 ± 0.8	*p* < 0.001	29 ± 0.2	32 ± 0.4	*p* < 0.001	29 ± 0.2	32.7 ± 1	*p* < 0.001	29.7 ± 0.5	31.3 ± 0.5	*p* < 0.001	
	HPRT1	29.9 ± 0.6	32.9 ± 0.7	*p* < 0.001	29.4 ± 0.4	32.3 ± 0.4	*p* < 0.001	29.9 ± 0.1	33.1 ± 0.9	*p* < 0.001	30.3 ± 0.5	32.2 ± 0.5	*p* < 0.001	

**Table 5.** Ct values of NFKB1, STAT3, IL6, TBP and HPRT1 genes obtained by our new method (50 µl of lysis solution) compared to TaqMan™ Cells-to-CT™ Express Kit from U-87 cells. Data are expressed as means ± s.d. (*n* =6). NEG, negative (non-stimulated); TNF1, control stimulated with TNFα 1 ng ml^−1^; TNF10, control stimulated with TNFα 10 ng ml^−1^; TNF100, control stimulated with TNFα 100 ng ml^−1^. ONM: our new method, RM: reference method (TaqMan™ Cells-to-CT™ Express Kit).

U-87		ONM Ct mean ± s.d.	RM Ct mean ± s.d.	*p*- value versus ONM	ONM Ct mean ± s.d.	RM Ct mean ± s.d.	*p*- value versus ONM	ONM Ct mean ± s.d.	RM Ct mean ± s.d.	*p-* value vs ONM	ONM Ct mean ± s.d.	RM Ct mean ± s.d.	*p*- value versus ONM
group		NEG			TNF1			TNF10			TNF100		
gene	IL6	27.4 ± 0.3	27.1 ± 0.7	*p* = 0.4	25.1 ± 0.1	24.9 ± 0.3	*p* = 0.2	24.7 ± 0.2	24.7 ± 0.5	*p* = 0.9	24.8 ± 0.5	24.4 ± 0.5	*p* = 0.2
	NFKB1	27.3 ± 0.2	29.4 ± 0.4	*p* < 0.001	26.5 ± 0.2	29.1 ± 0.4	*p* < 0.001	26.3 ± 0.2	29.3 ± 0.6	*p* < 0.001	26.5 ± 0.2	29 ± 0.3	*p* < 0.001
	STAT3	22.7 ± 0.1	24.8 ± 0.3	*p* < 0.001	22.1 ± 0.2	25.4 ± 0.6	*p* < 0.001	22.2 ± 0.2	25.2 ± 0.5	*p* < 0.001	22.6 ± 0.3	25.2 ± 0.2	*p* < 0.001
	TBP	26.8 ± 0.2	27.7 ± 0.6	*p* < 0.01	26.7 ± 0.2	28 ± 0.2	*p* < 0.001	26.6 ± 0.3	28.1 ± 0.2	*p* < 0.001	27 ± 0.1	27.9 ± 0.3	*p* < 0.001
	HPRT1	24.8 ± 0.2	25.9 ± 0.2	*p* < 0.001	24.3 ± 0.2	26 ± 0.1	*p* < 0.001	24.3 ± 0.2	26.1 ± 0.3	*p* < 0.001	24.5 ± 0.2	26 ± 0.2	*p* < 0.001

**Table 6.** Ct values of NFKB1, STAT3, IL6, TBP and HPRT1 genes obtained by our new method (25 µl of lysis solution) compared to TaqMan™ Cells-to-CT™ Express Kit from SK-HEP-1 cells. Data are expressed as means ± s.d. (*n* =6). NEG, negative (non-stimulated); TNF1, control stimulated with TNFα 1 ng ml^−1^; TNF10, control stimulated with TNFα 10 ng ml^−1^; TNF100, control stimulated with TNFα 100 ng ml^−1^. ONM, our new method, RM, reference method (TaqMan™ Cells-to-CT™ Express Kit).

SK-HEP-1		ONM Ct mean ± s.d.	RM Ct mean ± s.d.	*p*-value versus ONM	ONM Ct mean ± s.d.	RM Ct mean ± s.d.	*p*-value versus ONM	ONM Ct mean ± s.d.	RM Ct mean ± s.d.	*p*- value versus ONM	ONM Ct mean ± s.d.	RM Ct mean ± SD	*p*- value vs ONM	
group:		NEG			TNF1			TNF10			TNF100			
gene	IL6	26.1 ± 0.4	26.6 ± 0.7	*p* = 0.2	23.7 ± 0.3	24.1 ± 0.4	*p* = 0.2	23 ± 0.3	24.9 ± 0.4	*p* < 0.001	23 ± 0.1	25.7 ± 0.7	*p* < 0.01	
	NFKB1	25.8 ± 0.1	29.1 ± 0.6	*p* < 0.001	24.6 ± 0.2	27.4 ± 0.3	*p* < 0.001	24.1 ± 0.2	27.9 ± 0.2	*p* < 0.001	24 ± 0.1	28.8 ± 0.8	*p* < 0.001	
	STAT3	22.9 ± 0.1	26.5 ± 0.8	*p* < 0.001	22.3 ± 0.2	25 ± 0.4	*p* < 0.001	22.1 ± 0.1	25.6 ± 0.3	*p* < 0.001	22.1 ± 0.1	26.1 ± 0.5	*p* < 0.001	
	TBP	26.4 ± 0.3	27.9 ± 0.4	*p* < 0.001	26 ± 0.3	27.1 ± 0.2	*p* < 0.001	25.8 ± 0.2	28.2 ± 0.3	*p* < 0.001	25.7 ± 0.2	29.2 ± 0.7	*p* < 0.001	
	HPRT1	23.6 ± 0.2	25.6 ± 0.4	*p* < 0.001	23 ± 0.2	24.1 ± 0.4	*p* < 0.001	22.9 ± 0.2	26 ± 0.2	*p* < 0.001	23 ± 0.1	26.7 ± 0.4	*p* < 0.001	

Regarding ΔΔCt and RQ values, both methods showed comparable results (table 7). There was a significant difference between the non-stimulated group and the group comprising U87 cells stimulated with 100 ng ml^−1^ using the reference method for TNFα and STAT3 gene expression, while our new method did not show any significant difference. As the RQ differed ≤ 0.2, we do not consider this to have any practical significance. STAT3 expression also differed in the SK-HEP-1 cells in all three groups (cells stimulated with 1, 10 or 100 ng ml^−1^ TNFα) between our new method and the reference method. Our new method showed no significant difference between the non-stimulated group and the stimulated groups, but the reference method showed increased expression. As this increased expression was not observed with the Monarch® Total RNA Miniprep Kit, this difference must be attributed either to the different reverse transcription technique (e.g. reaching the upper limit of reverse transcription with the High-Capacity cDNA Reverse Transcription Kit) or to the pre-analytical phase (cell culture). In four instances, our new method showed a significant difference between the control group stimulated with LPS 100 ng ml^−1^ and the group pre-incubated with 100 µM of capsaicin or pre-incubated with 100 µM of hydroxychloroquine in PBMC cell cultures, compared to only one instance with the reference method. Additionally, in six instances, our new method showed a significant difference between the group stimulated with 1 ng ml^−1^ TNFα and the groups stimulated with 10 or 100 ng ml^−1^ TNFα in U-87 and SK-HEP-1 cells, compared to only two instances with the reference method.

**Table 7.** Comparison of ΔΔCt values and RQ values of NFKB1, STAT3 and IL6 genes obtained by our new method (PBMC, 50 µl of lysis solution; U-87, 25 µl of lysis solution; SK-HEP-1, 25 µl of lysis solution) and the TaqMan™ Cells-to-CT™ Express Kit from PBMC, U-87 and SK-HEP-1 cells. Data are expressed as means ± s.d. (*n* =6). NEG, negative (non-stimulated) control; CTR100, control stimulated with LPS 100 ng ml^−1^; CAP100, cells pre-incubated with 100 µM of capsaicin; HCQ 100, cells pre-incubated with 100 µM of hydroxychloroquine; TNF1, control stimulated with TNFα 1 ng ml^−1^; TNF10, control stimulated with TNFα 10 ng ml^−1^; TNF100, control stimulated with TNFα 100 ng ml^−1^. ONM, our new method, RM, reference method (TaqMan™ Cells-to-CT™ Express Kit). *p ≤ 0.05; **p ≤ 0.01; ***p ≤ 0.001 versus CTR100 or TNF1 group.

cell type	group	gene	ONM ΔΔCT mean ± s.d.	*p*-value versus NEG	ONM RQ	RM ΔΔCT mean ± s.d.	*p*-value versus NEG	RM RQ
PBMC	CTR100	IL6	−7.5 ± 0.9	*p* < 0.001	218	−6.5 ± 1.1	*p* < 0.001	114.5
		NFKB1	−1.6 ± 0.2	*p* < 0.001	3	−1.8 ± 0.7	*p* < 0.001	3.8
		STAT3	−0.9 ± 0.2	*p* < 0.001	1.9	−0.8 ± 0.5	*p* < 0.01	1.9
	CAP100	IL6	−9 ± 0.8*	*p* < 0.001	599.5	−7.5 ± 0.8	*p* < 0.001	205
		NFKB1	−1.9 ± 0.2*	*p* < 0.001	3.7	−2 ± 0.7	*p* < 0.001	4.6
		STAT3	−0.8 ± 0.1	*p* < 0.001	1.7	−1.1 ± 0.6	*p* < 0.01	2.3
	HCQ	IL6	−4.8 ± 0.8***	*p* < 0.001	31.6	−3.7 ± 0.9**	*p* < 0.001	14.9
		NFKB1	−1.2 ± 0.2*	*p* < 0.001	2.2	−1.6 ± 0.7	*p* < 0.001	3.3
		STAT3	−0.8 ± 0.1	*p* < 0.001	1.8	−0.6 ± 0.3	*p* < 0.001	1.6
U−87	TNF1	IL6	−1.9 ± 0.3	*p* < 0.001	3.9	−2.3 ± 0.4	*p* < 0.001	5.2
		NFKB1	−0.5 ± 0.2	*p* < 0.001	1.4	−0.6 ± 0.3	*p* < 0.001	1.5
		STAT3	−0.2 ± 0.3	*p* = 0.2	1.2	0.4 ± 0.6	*p* = 0.2	0.8
	TNF10	IL6	−2.3 ± 0.3*	*p* < 0.001	5.1	−2.6 ± 0.5	*p* < 0.001	6.6
		NFKB1	−0.6 ± 0.2	*p* < 0.001	1.6	−0.5 ± 0.4	*p* < 0.05	1.4
		STAT3	−0.1 ± 0.2	*p* = 0.4	1.1	0.1 ± 0.3	*p* = 0.5	1
	TNF100	IL6	−2.6 ± 0.2**	*p* < 0.001	6	−2.9 ± 0.8	*p* < 0.001	8.5
		NFKB1	−0.8 ± 0.2	*p* < 0.001	1.7	−0.6 ± 0.3	*p* < 0.01	1.6
		STAT3	0 ± 0.3	*p* = 0.3	1.1	0.2 ± 0.2	*p* < 0.05	0.9
SK-HEP-1	TNF1	IL6	−2 ± 0.2	*p* < 0.001	3.9	−1.8 ± 0.4	*p* < 0.001	3.5
		NFKB1	−0.7 ± 0.1	*p* < 0.001	1.7	−1 ± 0.6	*p* < 0.001	2.1
		STAT3	−0.1 ± 0.2	*p* < 0.001	1.1	−0.8 ± 0.6	*p* < 0.001	1.9
	TNF10	IL6	−2.5 ± 0.3**	*p* < 0.001	5.6	−2 ± 0.2	*p* < 0.001	4.1
		NFKB1	−1.1 ± 0.2**	*p* < 0.001	2.1	−1.6 ± 0.2*	*p* < 0.001	3.2
		STAT3	−0.2 ± 0.2	*p* < 0.05	1.2	−1.3 ± 0.3	*p* < 0.001	2.5
	TNF100	IL6	−2.5 ± 0.4**	*p* < 0.001	5.8	−2.1 ± 0.5	*p* < 0.001	4.6
		NFKB1	−1.1 ± 0.2**	*p* < 0.001	2.2	−1.6 ± 0.5	*p* < 0.001	3.3
		STAT3	−0.2 ± 0.2	*p* = 0.2	1.1	−1.7 ± 0.6*	*p* < 0.001	3.5

### 3.4. Fibroblast whole 96-well plate culture

**Figure 6.**
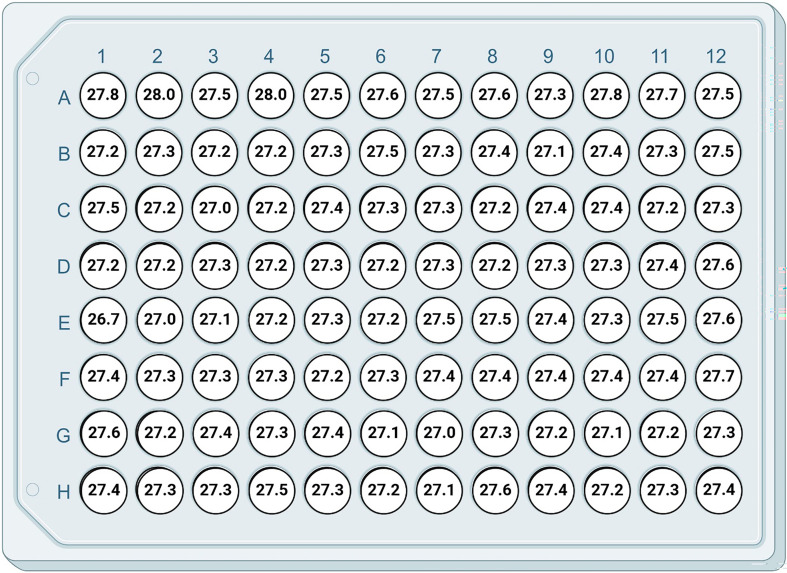
Mean Ct value of three housekeeping genes (HPRT1, YWHAZ and B2M) from 24 h fibroblast cell culture seeded at a density of 4 × 10^4^ cells ml^−1^.

To demonstrate the ability to process the whole 96-well plate, we used our new method of fibroblast cell culture seeded at every well of the 96-well plate at a density of 4 × 10^−4^ cells ^−1^. The mean Ct value of three housekeeping genes (HPRT1, YWHAZ and B2M) for each well is shown in figure 6. The mean ± s.d. of all 96 wells was 27.3 ± 0.2 with CV = at 0.7% indicating sufficient homogeneity.

### 3.5. DNase treatment

Adding DNase treatment before reverse transcription completely suppressed the signal from the no reverse transcriptase group when measuring the PPIA gene with gDNA amplifying assay. Unfortunately, the addition of DNase treatment significantly increased the mean Ct value (up to three cycles) in comparison with DNase-untreated groups.

### 3.6. day delayed reverse transcription

**Figure 7.**
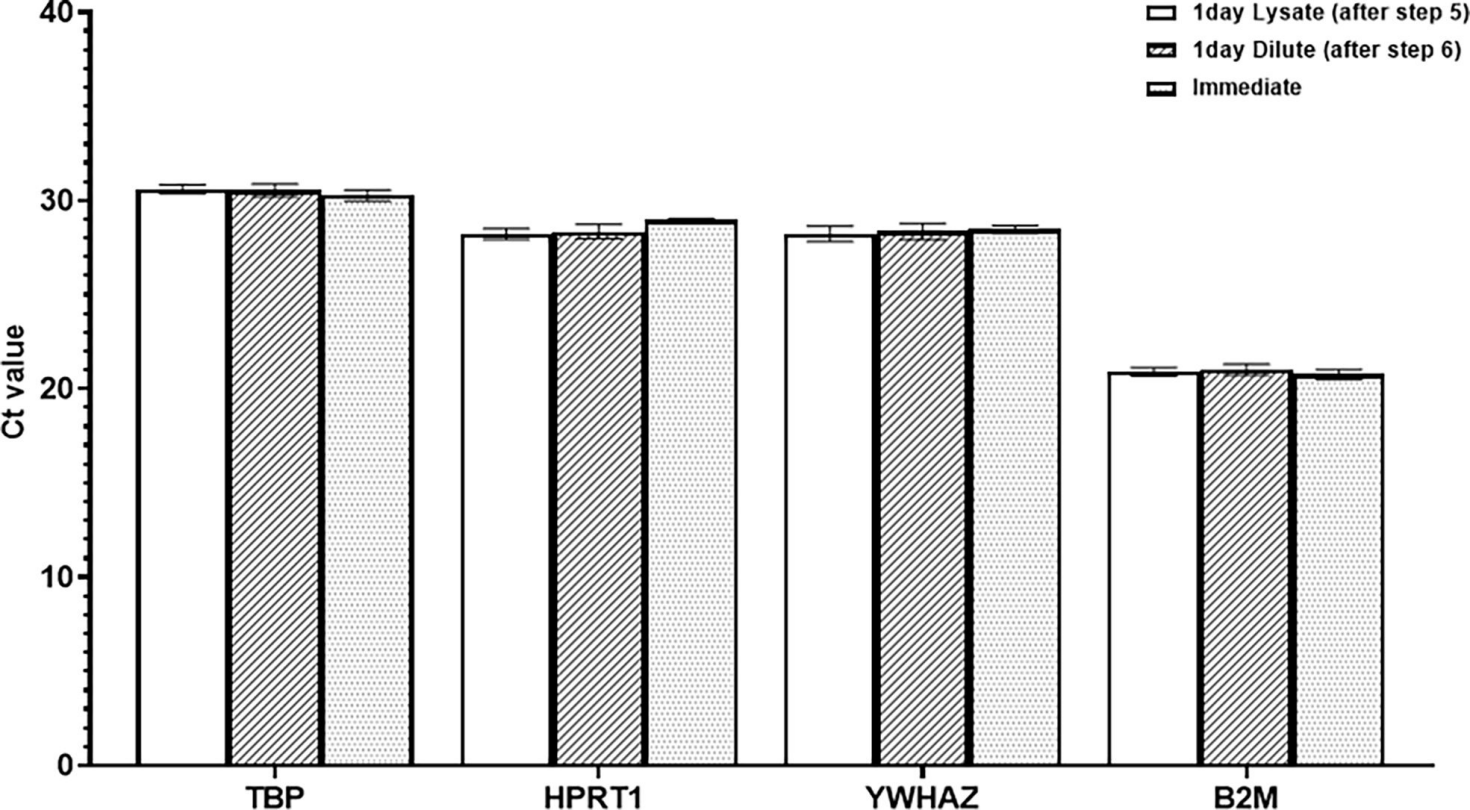
One day delayed reverse transcription: Ct values of four housekeeping genes (TBP, HPRT1, YWHAZ and B2M) obtained from non-stimulated PBMC. Data are expressed as mean ± s.d. (*n* = 6). Blank columns: reverse transcription after 1 day storage at −80°C continued from step 5 of our workflow; diagonal pattern columns: reverse transcription after 1 day storage at −80°C continued from step 6 of our workflow; dotted pattern columns: reverse transcription immediately the same day after step 6*.*

Gene expressions of four housekeeping genes (TBP, HPRT1, YWHAZ and B2M) were comparable (figure 7) when analysing samples from non-stimulated PBMCs, which were frozen after step 5 and step 6 in our simplified protocol (figure 1). Therefore, we conclude that reverse transcription could be postponed until the next day if samples are frozen at −80°C after step 5 (or step 6) in our simplified protocol (figure 1).

### 3.7. Cost comparison

**Figure 8.**
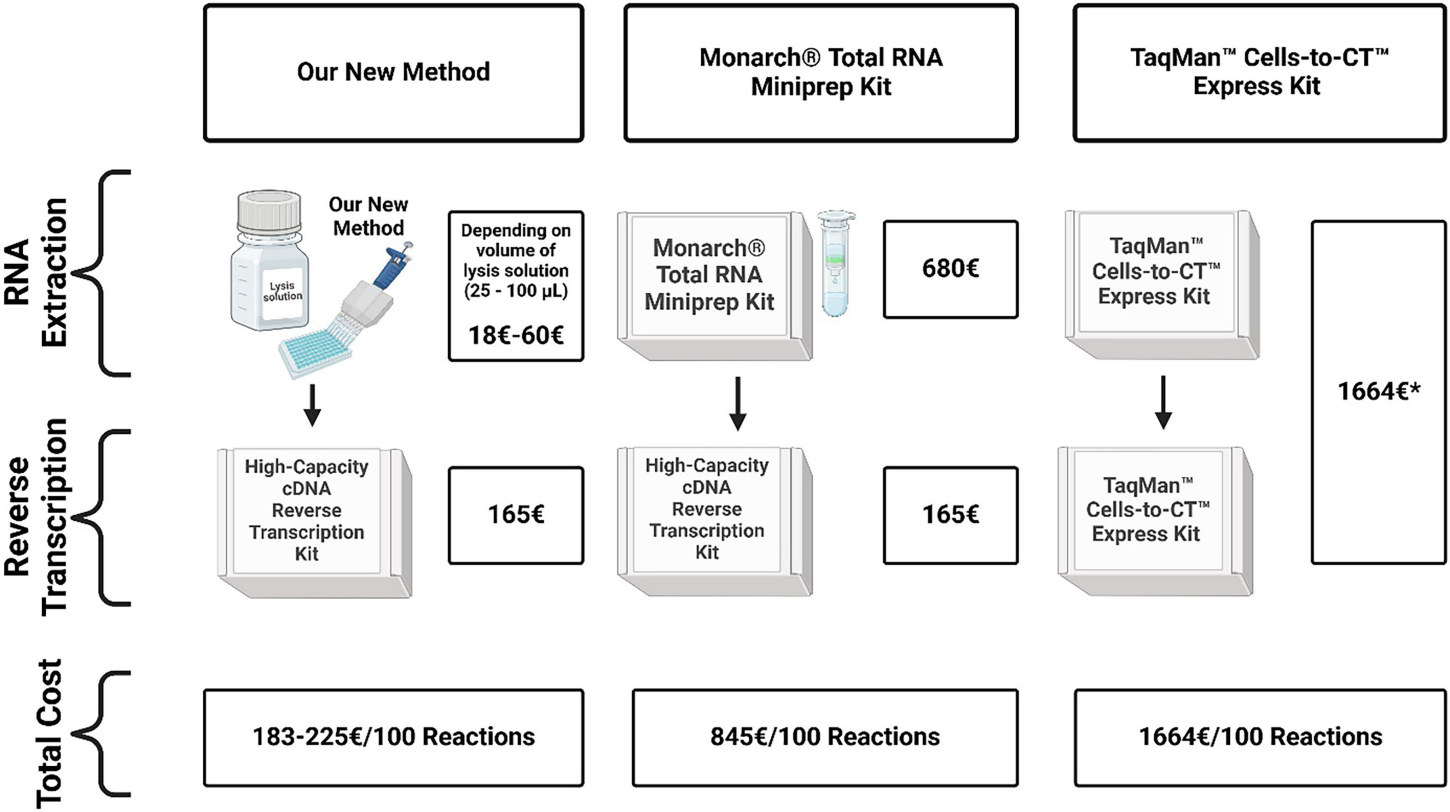
Breakdown of cost of our new method and reference methods. *Price of 5 ml of TaqMan™ Fast Advanced Master Mix included in reference kit (€95) was subtracted.

We compared the costs to synthetize cDNA using our new method with the reference methods (figure 8). Prices were obtained from official stores (Sigma-Aldrich; Thermo Fisher Scientific; New England Biolabs) as of July 2024. Prices do not include any sales or bulk discounts, shipping cost, taxes and are calculated for the minimum size product available for 100 reactions. If the minimum size product was sufficient for more than 100 reactions, the price of the product was divided according to the price obtained for 100 reactions. As the TaqMan™ Cells-to-CT™ Express Kit contains TaqMan™ Fast Advanced Master Mix, the price of equally sized separately sold TaqMan™ Fast Advanced Master Mix was subtracted. In conclusion, the price for 100 reactions to synthetize cDNA for our new method ranged from €183 when 25 µl of lysis solution was used to €225 when 100 µl of lysis solution was used. In comparison, the total cost of 100 reactions to synthetize cDNA using the Monarch® Total RNA Miniprep Kit was €845 (4.6–3.8 times higher than our new method) and the total cost of 100 reactions to synthetize cDNA using the TaqMan™ Cells-to-CT™ Express Kit was €1664 (9.1–7.4 times higher).

## 4. Discussion

The development of our new method was inspired by previously described protocols in the literature by Bender *et al*. [5] and Goldenberg *et al*. [6]. Our newly developed method uses SDS, DDT and proteinase K for RNase denaturation and degradation. Subsequently, the denaturation effect of SDS is neutralized by the dilution of the lysed sample with a Tween 20 solution. After a series of experiments, we established the most optimal concentrations of SDS (0.5%), DTT (10 mM) and proteinase K (1 mg ml^−1^). Other concentrations of DDT and proteinase K in the lysis solution, either higher or lower, showed unsatisfactory results. We have also tested different amounts of lysis solution, different concentrations of Tween 20 solution and different ratios of dilution of lysed samples in Tween 20 solution. The amount of lysis solution can be adjusted to the specific cell type and number of cells. A dilution as low as two times proved to be fully functional and compatible with the reverse transcription kit we used. With two times dilution, a 20% Tween 20 solution proved to be optimal in terms of variability and Ct value.

We also tried different approaches based on methods found in the literature, namely the works of Shatzkes *et al*. [7], Le *et al*. [8] and Svec *et al*. [9]. These approaches are based either on detecting viral RNA or RNA from a low number of cells. Unfortunately, we were unable to scale these methods for our cell culture. It is possible that this failure was due to the high content of RNase present in cell lysates, thus requiring more potent protection for present mRNA.

We have chosen the TaqMan™ Cells-to-CT™ Express Kit as the reference method, which is based on the same principle as ours (i.e. ‘Cells-to-cDNA’ approach) and can process a whole 96-well plate with up to 10^5^ cells per well. From RNA purification methods, we selected the spin column-based Monarch ® Total RNA Miniprep Kit. The spin column method is a widely used standard method for RNA purification that is nontoxic, provides a high yield of extracted RNA, and is adaptable for 96-well plate processing [10].

Regarding the cDNA yield, with the Monarch ® Total RNA Miniprep Kit, cDNA was effectively diluted in 200 µl volume (100 µl of elution solution + 2 × dilution in reverse transcription), with the TaqMan™ Cells-to-CT™ Express Kit cDNA was effectively diluted in 200 µl volume (50 µl lysis solution + 4 × dilution in reverse transcription) and with our new method cDNA was effectively diluted in 100 µl volume for 25 µl lysis solution, in 200 µl volume for 50 µl lysis solution and in 400 µl for 100 µl lysis solution (volume of lysis solution + 2 × dilution in Tween 20 + 2 × dilution in reverse transcription). In the case of PBMC cells, we can conclude that the cDNA yield was clearly higher with our new method as cDNA in our new method was effectively diluted in the same or higher volume than with the reference method. The same could be stated in the case of U-87 cells in comparison to the TaqMan™ Cells-to-CT™ Express Kit. In the case of U-87 cells compared to the Monarch ® Total RNA Miniprep Kit and in the case of SK-HEP-1 cells versus both reference methods, it is less straightforward as we used effectively two times more concentrated cDNA. But as the difference in Ct values was higher than 1 (value expected with two times dilution) we can conclude that probably there is also a higher cDNA yield.

Our results clearly show a higher cDNA yield using our new method compared with the reference commercial methods. Although we have not tested any other reverse transcription kits, we expect full compatibility since the method works with one of the simplest and cheapest options on the market.

Our new method demonstrated improved sensitivity in detecting gene expression differences over the reference method, as shown by the effect of capsaicin pre-treatment, which should enhance the LPS-induced pro-inflammatory response, e.g. increased IL6 and NFKB1 gene expression [11–13]. The TaqMan™ Cells-to-CT™ Express Kit failed to show this effect of capsaicin, while our new method successfully demonstrated it. Additionally, our new method better distinguishes differences in gene expressions between groups treated with increasing doses of TNFα in U-87 and SK-HEP-1 cells. This improved sensitivity is likely due to the lower observed variability in our new method compared with the reference methods.

The processing time prior to the reverse transcription is less demanding in terms of manual labour compared with the Monarch® Total RNA Miniprep Kit, with approximately 10 min of manual labour followed by 1 h incubation using our new method compared with approximately 1 h of manual labour with the reference method. In addition, our new method generates less waste because there are fewer pipetting steps, as the spin column methods involve washing and elution steps thus requiring additional tubes and pipette tips. Our method only needs one additional PCR plate. Although the time to synthetize cDNA using our new method appears to be much longer (approx. 2.5 h) compared with the TaqMan™ Cells-to-CT™ Express Kit, the commercial kit uses an advanced reverse transcriptase enzyme that reduces the time for the reverse transcription reaction to only 20 min. Therefore, if we only count the processing time prior to the reverse transcription, the actual manual work time is similar to around 10 min for both methods.

There is a clear economic advantage to our new method. When comparing the cost of 100 reactions from sample to cDNA using our new method with the TaqMan™ Cells-to-CT™ Express Kit and the Monarch® Total RNA Miniprep Kit, our new method costs are reduced almost to one-tenth and one-fifth, respectively. Of course, with sales, bulk orders or sourcing from different suppliers, the price for all three methods could be reduced. Nevertheless, we believe that our basic comparison of 100 reactions demonstrates the cost rations between all three methods most clearly. Regarding the labour cost, as discussed in the previous paragraph, the actual manual work time is similar, around 10 min for our new method and the TaqMan™ Cells-to-CT™ Express Kit, compared with approximately 1 h with the Monarch® Total RNA Miniprep Kit. As our new method includes only simple pipetting steps, we believe it could also be adapted for automation.

The main disadvantages of our new method are incompatibility with SYBR Green-based qPCR and with DNase treatment, which requires cDNA-specific probes and primers. A summary of the key advantages and disadvantages is presented in table 8.

**Table 8.** Key advantages and disadvantages of our new method.

advantages	disadvantages
higher cDNA yield compared to reference methods	SYBR green incompatible
simple workflow	DNase incompatible
easy streamlining	—
much cheaper compared with reference methods	—
more sensitive, lower variability compared with reference methods	—
fully compatible with probe-based qPCR	—
no special equipment needed	—
no toxic substances	—
less laboratory waste compared to reference methods	—

## 5. Conclusion

In conclusion, we have developed a new, simple, reliable and streamlined method for cDNA synthesis from cell culture lysates that allows low workload processing of 96-well cell culture plates at drastically reduced costs. Moreover, the method possesses superior sensitivity and lower variability compared with the commercially available reference methods. This method is optimal for the screening of multiple compounds and concentrations, making it applicable in processes such as drug development.

## Data Availability

The data can be accessed at the public data repository OSF via the following link: https://osf.io/39qnh/?view_only=82e8a5f7d2b44058b6975cb5cb52539e [14].
